# K_2_LaCl_5_

**DOI:** 10.1107/S1600536810045198

**Published:** 2010-11-10

**Authors:** Christian M. Schurz, Thomas Schleid, Gerd Meyer

**Affiliations:** a Institut für Anorganische Chemie, Universität Stuttgart, Pfaffenwaldring 55, 70569 Stuttgart, Germany; b Department für Chemie, Institut für Anorganische Chemie, Universität zu Köln, Greinstrasse 6, 50939 Köln, Germany

## Abstract

The ternary title compound, dipotassium lanthanum penta­chloride, K_2_LaCl_5_, is isotypic with Y_2_HfS_5_ and various ternary rare-earth metal(III) halides with the general formula *A*_2_ *MX*_5_ (*A* = NH_4_, In^I^, Na–Cs; *M* = La–Dy; *X* = Cl–I). The La^3+^ cations and three of the four symmetry-independent chloride anions are located on a crystallographic mirror plane. The La^3+^ cations are surrounded by seven chloride anions, each in the shape of a monocapped trigonal prism, whereas the coordination spheres of the K^+^ cations exhibit one more cap. Three of the four independent chloride anions reside in a fivefold cationic coordination, leading to distorted square pyramids. The fourth chloride anion has only four cationic neighbours, forming no specific polyhedron.

## Related literature

For the U_3_ *Ch*_5_-type structure (*Ch* = S and Se) and its relationship to Y_2_HfS_5_, see: Moseley *et al.* (1972 ▶); Potel *et al.* (1972 ▶); Jeitschko & Donohue (1975 ▶). For the low-temperature phase of Yb_5_Sb_3_, see: Brunton & Steinfink (1971 ▶). For the series of the ternary rare-earth metal(III) halides with *A* = NH_4_, In^I^, Na – Cs; *M* = La – Dy; *X* = Cl – I, see: Meyer & Hüttl (1983 ▶); Meyer *et al.* (1985 ▶); Wickleder & Meyer (1995 ▶).

## Experimental

### 

#### Crystal data

K_2_LaCl_5_
*M* *_r_* = 394.36
Orthorhombic, 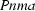

*a* = 12.7402 (8) Å
*b* = 8.8635 (6) Å
*c* = 8.0174 (5) Å
*V* = 905.35 (10) Å^3^
*Z* = 4
Mo *K*α radiation
μ = 7.02 mm^−1^
*T* = 293 K
0.33 × 0.28 × 0.24 mm

#### Data collection

Stoe IPDS-I diffractometer
Absorption correction: numerical (*X-SHAPE*; Stoe & Cie, 1999 ▶) *T*_min_ = 0.106, *T*_max_ = 0.185
12421 measured reflections
1650 independent reflections
872 reflections with *I* > 2σ(*I*)
*R*_int_ = 0.139

#### Refinement

*R*[*F* ^2^ > 2σ(*F* ^2^)] = 0.059
*wR*(*F* ^2^) = 0.142
*S* = 0.90
1650 reflections
44 parameters
Δρ_max_ = 1.58 e Å^−3^
Δρ_min_ = −2.64 e Å^−3^

### 

Data collection: *DIF4* (Stoe & Cie, 1992 ▶); cell refinement: *DIF4*; data reduction: *REDU4* (Stoe & Cie, 1992 ▶); program(s) used to solve structure: *SHELXS97* (Sheldrick, 2008 ▶); program(s) used to refine structure: *SHELXL97* (Sheldrick, 2008 ▶); molecular graphics: *DIAMOND* (Brandenburg, 2006 ▶); software used to prepare material for publication: *SHELXL97*.

## Supplementary Material

Crystal structure: contains datablocks I, global. DOI: 10.1107/S1600536810045198/bt5401sup1.cif

Structure factors: contains datablocks I. DOI: 10.1107/S1600536810045198/bt5401Isup2.hkl

Additional supplementary materials:  crystallographic information; 3D view; checkCIF report

## Figures and Tables

**Table 1:** Selected bond lengths (Å)

K—Cl1^i^	3.160 (3)
K—Cl2	3.177 (3)
K—Cl1^ii^	3.206 (3)
K—Cl2^iii^	3.234 (3)
K—Cl3^iv^	3.272 (4)
K—Cl4	3.304 (3)
K—Cl4^iii^	3.327 (3)
K—Cl3	3.351 (4)
La—Cl3^v^	2.812 (3)
La—Cl1^i^	2.833 (3)
La—Cl2^vi^	2.845 (3)
La—Cl4	2.858 (2)
La—Cl4^vii^	2.858 (2)
La—Cl4^viii^	2.895 (2)
La—Cl4^ix^	2.895 (2)

Symmetry codes: (i) 
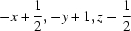
; (ii) 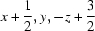; (iii) 
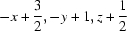
; (iv) 
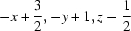
; (v) 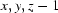; (vi) 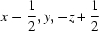; (vii) 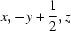; (viii) 
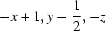
; (ix) 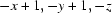.

**Fig. 1.**
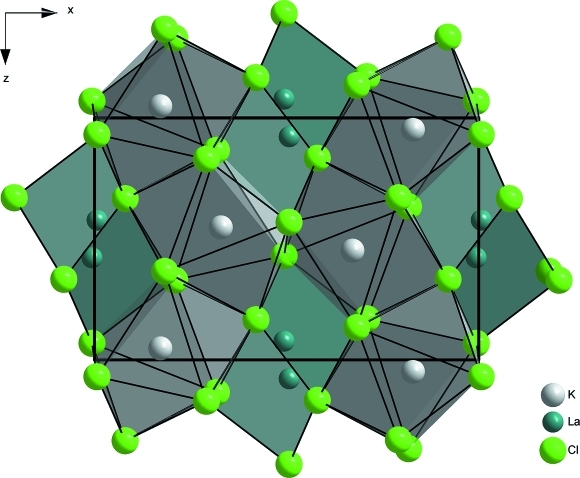
Crystal structure of K2LaCl5 as viewed along [010].

**Fig. 2.**
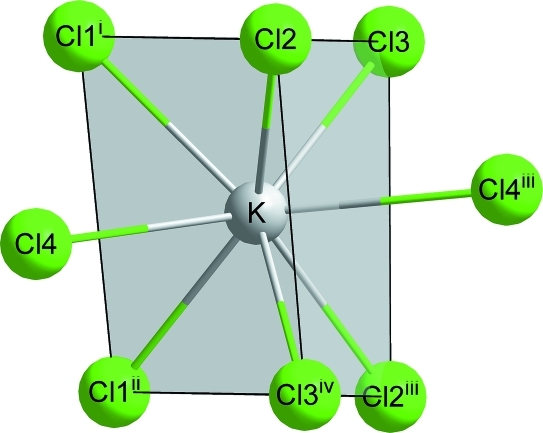
Coordination sphere of the K+ cations with the shape of a bicapped trigonal prism. [Symmetry codes: (i) –x+1/2, –y + 1, z–1/2; (ii) x+1/2, y, –z+3/2; (iii) –x+3/2, –y + 1, z+1/2; (iv) –x+3/2, –y + 1, z–1/2.]

**Fig. 3.**
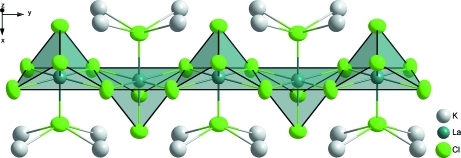
View at the chain formed by edge-sharing monocapped trigonal prisms [LaCl7]4– with its contacts to the K+ cations. Displacement ellipsoids are drawn at 90% probability level.

## supplementary crystallographic information

### Comment

The ternary rare-earth metal(III) halide K_2_LaCl_5_ (Fig. 1) belongs to the *A*_2_*MX*_5_ series (*A* = NH_4_, In, Na – Cs; *M* = La – Dy; *X* = Cl – I) (Meyer & Hüttl, 1983; Meyer *et al.*, 1985; Wickleder & Meyer 1995).  It can be described as ordered structural variety of U_3_*Ch*_5_ (*Ch* = S and Se) or the low-temperature phase of Yb_5_Sb_3_, respectively, as anti-isotypical arrangement. While the K^+^ cations have eight contacts to Cl^-^ anions (Fig. 2), the La^3+^ cations are surrounded by only seven of them. In both cases distorted mono- or bicapped trigonal prisms [LaCl_7_]^4–^ or [KCl_8_]^7–^ originate. For the lanthanum bearing ones they are linked *via* common edges and form chains, which run along [010] (Fig. 3). Together with the chloride anions (Cl1)^-^, (Cl2)^– ^and (Cl3)^-^, La^3+^ occupies the 4*c* position and shows the site symmetry *m*, while the (Cl4)^-^ anion and the K^+^ cation are located at the 8*d* position with the site symmetry 1.

### Experimental

Colourless, transparent, brick-shaped single crystals of K_2_LaCl_5_ were obtained as by-product from the reaction of potassium azide (KN_3_), lanthanum (La), the corresponding sesquioxide (La_2_O_3_) and trichloride (LaCl_3_) in the presence of KCl as flux with the purpose to synthesize K_2_La_4_ONCl_9_. The mixture was transferred into a torch-sealed, evacuated, fused silica vessel, heated at 1123 K for seven days, followed by cooling to room temperature within 24 h.

### Crystal data

**Table:**

K_2_LaCl_5_	*F*(000) = 720
*M**_r_* = 394.36	*D*_x_ = 2.893 Mg m^−^^3^
Orthorhombic, *P**n**m**a*	Mo *K*α radiation, λ = 0.71073 Å
Hall symbol: -P 2ac 2n	θ = 3.4–33.0°
*a* = 12.7402 (8) Å	µ = 7.02 mm^−^^1^
*b* = 8.8635 (6) Å	*T* = 293 K
*c* = 8.0174 (5) Å	Bricks, colourless
*V* = 905.35 (10) Å^3^	0.33 × 0.28 × 0.24 mm
*Z* = 4	

### Data collection

**Table:**

Stoe IPDS-I diffractometer	1650 independent reflections
Radiation source: fine-focus sealed tube	872 reflections with *I* > 2σ(*I*)
graphite	*R*_int_ = 0.139
imaging plate detector system scans	θ_max_ = 33.0°, θ_min_ = 3.4°
Absorption correction: numerical (*X-SHAPE*; Stoe & Cie, 1999)	*h* = −19→19
*T*_min_ = 0.106, *T*_max_ = 0.185	*k* = −11→11
12421 measured reflections	*l* = −12→12

### Refinement

**Table:**

Refinement on *F*^2^	Primary atom site location: structure-invariant direct methods
Least-squares matrix: full	Secondary atom site location: difference Fourier map
*R*[*F*^2^ > 2σ(*F*^2^)] = 0.059	*w* = 1/[σ^2^(*F*_o_^2^) + (0.0799*P*)^2^] where *P* = (*F*_o_^2^ + 2*F*_c_^2^)/3
*wR*(*F*^2^) = 0.142	(Δ/σ)_max_ = 0.004
*S* = 0.90	Δρ_max_ = 1.58 e Å^−^^3^
1650 reflections	Δρ_min_ = −2.64 e Å^−^^3^
44 parameters	Extinction correction: *SHELXL97* (Sheldrick, 2008), F_c_^*^=kF_c_[1+0.001xF_c_^2^λ^3^/sin(2θ)]^-1/4^
0 restraints	Extinction coefficient: 0.0094 (12)

### Special details

**Table:**

Geometry. All e.s.d.'s (except the e.s.d. in the dihedral angle between two l.s. planes) are estimated using the full covariance matrix. The cell e.s.d.'s are taken into account individually in the estimation of e.s.d.'s in distances, angles and torsion angles; correlations between e.s.d.'s in cell parameters are only used when they are defined by crystal symmetry. An approximate (isotropic) treatment of cell e.s.d.'s is used for estimating e.s.d.'s involving l.s. planes.
Refinement. Refinement of *F*^2^ against ALL reflections. The weighted *R*-factor *wR* and goodness of fit *S* are based on *F*^2^, conventional *R*-factors *R* are based on *F*, with *F* set to zero for negative *F*^2^. The threshold expression of *F*^2^ > σ(*F*^2^) is used only for calculating *R*-factors(gt) *etc*. and is not relevant to the choice of reflections for refinement. *R*-factors based on *F*^2^ are statistically about twice as large as those based on *F*, and *R*- factors based on ALL data will be even larger.

### Fractional atomic coordinates and isotropic or equivalent isotropic displacement parameters (Å^2^)

**Table:**

	*x*	*y*	*z*	*U*_iso_*/*U*_eq_	
K	0.67125 (15)	0.4946 (3)	0.5481 (3)	0.0379 (5)	
La	0.50680 (5)	0.2500	0.07776 (8)	0.0248 (2)	
Cl1	−0.0065 (2)	0.7500	0.9311 (4)	0.0310 (6)	
Cl2	0.7911 (2)	0.2500	0.3299 (4)	0.0333 (7)	
Cl3	0.6828 (2)	0.2500	0.8662 (4)	0.0374 (8)	
Cl4	0.57990 (17)	0.5441 (3)	0.1663 (3)	0.0342 (5)	

### Atomic displacement parameters (Å^2^)

**Table:**

	*U*^11^	*U*^22^	*U*^33^	*U*^12^	*U*^13^	*U*^23^
K	0.0381 (10)	0.0363 (14)	0.0393 (11)	0.0014 (8)	−0.0014 (7)	−0.0070 (9)
La	0.0286 (3)	0.0234 (4)	0.0222 (3)	0.000	0.0022 (3)	0.000
Cl1	0.0347 (13)	0.0354 (17)	0.0228 (11)	0.000	0.0016 (12)	0.000
Cl2	0.0282 (13)	0.039 (2)	0.0322 (15)	0.000	0.0006 (11)	0.000
Cl3	0.0368 (15)	0.045 (2)	0.0308 (15)	0.000	0.0088 (12)	0.000
Cl4	0.0460 (12)	0.0267 (13)	0.0301 (10)	−0.0030 (9)	−0.0102 (8)	0.0025 (8)

### Geometric parameters (Å, °)

**Table:**

K—Cl1^i^	3.160 (3)	La—Cl4^x^	2.895 (2)
K—Cl2	3.177 (3)	La—K^vi^	4.389 (2)
K—Cl1^ii^	3.206 (3)	La—K^xi^	4.389 (2)
K—Cl2^iii^	3.234 (3)	Cl1—La^xii^	2.833 (3)
K—Cl3^iv^	3.272 (4)	Cl1—K^xii^	3.160 (3)
K—Cl4	3.304 (3)	Cl1—K^xiii^	3.160 (3)
K—Cl4^iii^	3.327 (3)	Cl1—K^xiv^	3.206 (3)
K—Cl3	3.351 (4)	Cl1—K^xv^	3.206 (3)
K—K^v^	4.336 (5)	Cl2—La^xvi^	2.845 (3)
K—La^vi^	4.389 (2)	Cl2—K^v^	3.177 (3)
K—K^vi^	4.432 (4)	Cl2—K^xvii^	3.234 (3)
K—K^iii^	4.4838 (18)	Cl2—K^iv^	3.234 (3)
La—Cl3^vii^	2.812 (3)	Cl3—La^xviii^	2.812 (3)
La—Cl1^i^	2.833 (3)	Cl3—K^iii^	3.272 (4)
La—Cl2^viii^	2.845 (3)	Cl3—K^xix^	3.272 (3)
La—Cl4	2.858 (2)	Cl3—K^v^	3.351 (4)
La—Cl4^v^	2.858 (2)	Cl4—La^x^	2.895 (2)
La—Cl4^ix^	2.895 (2)	Cl4—K^iv^	3.327 (3)
			
Cl1^i^—K—Cl2	71.80 (8)	Cl3^vii^—La—Cl4	83.68 (6)
Cl1^i^—K—Cl1^ii^	91.76 (5)	Cl1^i^—La—Cl4	75.64 (5)
Cl2—K—Cl1^ii^	148.21 (10)	Cl2^viii^—La—Cl4	104.48 (5)
Cl1^i^—K—Cl2^iii^	141.81 (10)	Cl3^vii^—La—Cl4^v^	83.68 (6)
Cl2—K—Cl2^iii^	142.29 (7)	Cl1^i^—La—Cl4^v^	75.64 (5)
Cl1^ii^—K—Cl2^iii^	64.80 (8)	Cl2^viii^—La—Cl4^v^	104.48 (5)
Cl1^i^—K—Cl3^iv^	136.03 (10)	Cl4—La—Cl4^v^	131.62 (9)
Cl2—K—Cl3^iv^	87.34 (7)	Cl3^vii^—La—Cl4^ix^	84.06 (7)
Cl1^ii^—K—Cl3^iv^	86.34 (8)	Cl1^i^—La—Cl4^ix^	132.50 (6)
Cl2^iii^—K—Cl3^iv^	75.11 (8)	Cl2^viii^—La—Cl4^ix^	78.89 (7)
Cl1^i^—K—Cl4	65.30 (7)	Cl4—La—Cl4^ix^	150.15 (6)
Cl2—K—Cl4	75.52 (8)	Cl4^v^—La—Cl4^ix^	73.58 (7)
Cl1^ii^—K—Cl4	72.88 (8)	Cl3^vii^—La—Cl4^x^	84.06 (7)
Cl2^iii^—K—Cl4	127.34 (10)	Cl1^i^—La—Cl4^x^	132.50 (6)
Cl3^iv^—K—Cl4	72.27 (8)	Cl2^viii^—La—Cl4^x^	78.89 (7)
Cl1^i^—K—Cl4^iii^	130.30 (10)	Cl4—La—Cl4^x^	73.58 (7)
Cl2—K—Cl4^iii^	68.18 (8)	Cl4^v^—La—Cl4^x^	150.15 (6)
Cl1^ii^—K—Cl4^iii^	136.98 (9)	Cl4^ix^—La—Cl4^x^	78.15 (9)
Cl2^iii^—K—Cl4^iii^	74.46 (8)	Cl3^vii^—La—K^vi^	145.53 (4)
Cl3^iv^—K—Cl4^iii^	69.93 (8)	Cl1^i^—La—K^vi^	46.84 (5)
Cl4—K—Cl4^iii^	127.83 (9)	Cl2^viii^—La—K^vi^	47.41 (5)
Cl1^i^—K—Cl3	79.12 (8)	Cl4—La—K^vi^	61.85 (6)
Cl2—K—Cl3	87.50 (8)	Cl4^v^—La—K^vi^	117.97 (6)
Cl1^ii^—K—Cl3	116.64 (9)	Cl4^ix^—La—K^vi^	126.15 (5)
Cl2^iii^—K—Cl3	85.10 (7)	Cl4^x^—La—K^vi^	86.55 (6)
Cl3^iv^—K—Cl3	139.63 (8)	Cl3^vii^—La—K^xi^	145.53 (4)
Cl4—K—Cl3	143.76 (10)	Cl1^i^—La—K^xi^	46.84 (5)
Cl4^iii^—K—Cl3	71.00 (8)	Cl2^viii^—La—K^xi^	47.41 (5)
Cl1^i^—K—K^v^	46.68 (5)	Cl4—La—K^xi^	117.97 (6)
Cl2—K—K^v^	46.96 (6)	Cl4^v^—La—K^xi^	61.85 (6)
Cl1^ii^—K—K^v^	134.91 (5)	Cl4^ix^—La—K^xi^	86.55 (6)
Cl2^iii^—K—K^v^	134.42 (6)	Cl4^x^—La—K^xi^	126.15 (6)
Cl3^iv^—K—K^v^	133.77 (6)	K^vi^—La—K^xi^	62.09 (7)
Cl4—K—K^v^	97.63 (6)	La^xii^—Cl1—K^xii^	107.21 (8)
Cl4^iii^—K—K^v^	84.07 (6)	La^xii^—Cl1—K^xiii^	107.21 (8)
Cl3—K—K^v^	49.68 (5)	K^xii^—Cl1—K^xiii^	86.64 (11)
Cl1^i^—K—La^vi^	102.32 (7)	La^xii^—Cl1—K^xiv^	93.04 (7)
Cl2—K—La^vi^	167.59 (9)	K^xii^—Cl1—K^xiv^	159.73 (10)
Cl1^ii^—K—La^vi^	40.13 (5)	K^xiii^—Cl1—K^xiv^	88.24 (5)
Cl2^iii^—K—La^vi^	40.37 (6)	La^xii^—Cl1—K^xv^	93.04 (7)
Cl3^iv^—K—La^vi^	103.97 (7)	K^xii^—Cl1—K^xv^	88.24 (5)
Cl4—K—La^vi^	112.49 (7)	K^xiii^—Cl1—K^xv^	159.73 (10)
Cl4^iii^—K—La^vi^	110.55 (6)	K^xiv^—Cl1—K^xv^	89.82 (11)
Cl3—K—La^vi^	80.57 (6)	La^xvi^—Cl2—K^v^	108.72 (9)
K^v^—K—La^vi^	121.04 (3)	La^xvi^—Cl2—K	108.72 (9)
Cl1^i^—K—K^vi^	46.30 (6)	K^v^—Cl2—K	86.07 (11)
Cl2—K—K^vi^	113.09 (10)	La^xvi^—Cl2—K^xvii^	92.22 (8)
Cl1^ii^—K—K^vi^	45.45 (6)	K^v^—Cl2—K^xvii^	88.75 (3)
Cl2^iii^—K—K^vi^	104.62 (9)	K—Cl2—K^xvii^	159.00 (11)
Cl3^iv^—K—K^vi^	117.84 (10)	La^xvi^—Cl2—K^iv^	92.22 (8)
Cl4—K—K^vi^	59.28 (6)	K^v^—Cl2—K^iv^	159.00 (11)
Cl4^iii^—K—K^vi^	171.90 (11)	K—Cl2—K^iv^	88.75 (3)
Cl3—K—K^vi^	100.93 (9)	K^xvii^—Cl2—K^iv^	88.84 (11)
K^v^—K—K^vi^	91.23 (7)	La^xviii^—Cl3—K^iii^	100.62 (8)
La^vi^—K—K^vi^	66.36 (5)	La^xviii^—Cl3—K^xix^	100.62 (8)
Cl1^i^—K—K^iii^	125.25 (10)	K^iii^—Cl3—K^xix^	87.54 (11)
Cl2—K—K^iii^	106.95 (10)	La^xviii^—Cl3—K	115.09 (9)
Cl1^ii^—K—K^iii^	104.75 (8)	K^iii^—Cl3—K	85.21 (4)
Cl2^iii^—K—K^iii^	45.10 (6)	K^xix^—Cl3—K	144.27 (10)
Cl3^iv^—K—K^iii^	97.44 (9)	La^xviii^—Cl3—K^v^	115.10 (9)
Cl4—K—K^iii^	169.44 (11)	K^iii^—Cl3—K^v^	144.27 (10)
Cl4^iii^—K—K^iii^	47.24 (6)	K^xix^—Cl3—K^v^	85.21 (4)
Cl3—K—K^iii^	46.65 (6)	K—Cl3—K^v^	80.64 (10)
K^v^—K—K^iii^	91.22 (6)	La—Cl4—La^x^	106.42 (7)
La^vi^—K—K^iii^	67.00 (4)	La—Cl4—K	102.93 (8)
K^vi^—K—K^iii^	126.56 (8)	La^x^—Cl4—K	147.62 (10)
Cl3^vii^—La—Cl1^i^	127.18 (9)	La—Cl4—K^iv^	98.37 (8)
Cl3^vii^—La—Cl2^viii^	157.97 (10)	La^x^—Cl4—K^iv^	103.65 (8)
Cl1^i^—La—Cl2^viii^	74.85 (9)	K—Cl4—K^iv^	85.10 (6)

Symmetry codes: (i) −*x*+1/2, −*y*+1, *z*−1/2; (ii) *x*+1/2, *y*, −*z*+3/2; (iii) −*x*+3/2, −*y*+1, *z*+1/2; (iv) −*x*+3/2, −*y*+1, *z*−1/2; (v) *x*, −*y*+1/2, *z*; (vi) −*x*+1, −*y*+1, −*z*+1; (vii) *x*, *y*, *z*−1; (viii) *x*−1/2, *y*, −*z*+1/2; (ix) −*x*+1, *y*−1/2, −*z*; (x) −*x*+1, −*y*+1, −*z*; (xi) −*x*+1, *y*−1/2, −*z*+1; (xii) −*x*+1/2, −*y*+1, *z*+1/2; (xiii) −*x*+1/2, *y*+1/2, *z*+1/2; (xiv) *x*−1/2, −*y*+3/2, −*z*+3/2; (xv) *x*−1/2, *y*, −*z*+3/2; (xvi) *x*+1/2, *y*, −*z*+1/2; (xvii) −*x*+3/2, *y*−1/2, *z*−1/2; (xviii) *x*, *y*, *z*+1; (xix) −*x*+3/2, *y*−1/2, *z*+1/2.
